# Enhancing cognitive function in older adults: dietary approaches and implications

**DOI:** 10.3389/fnut.2024.1286725

**Published:** 2024-01-31

**Authors:** Baruh Polis, Abraham O. Samson

**Affiliations:** Laboratory of Computational Biology and Drug Discovery, Azrieli Faculty of Medicine, Bar Ilan University, Safed, Israel

**Keywords:** aging, senile dementia, protein malnutrition, diet, branched-chain amino acids

## Abstract

Natural aging encompasses physiological and psychological changes that impact overall health and quality of life. Mitigating these effects requires physical and mental exercise, coupled with proper nutrition. Notably, protein malnutrition emerges as a potential risk factor for senile dementia, with insufficient intake correlating with premature cognitive decline. Adequate protein intake in the elderly positively associates with memory function and lowers cognitive impairment risk. Considering diet as a modifiable risk factor for cognitive decline, extensive research has explored diverse dietary strategies to prevent dementia onset in older adults. However, conclusive results remain limited. This review aims to synthesize recent evidence on effective dietary approaches to enhance cognitive function and prognosis in older individuals. Specifically, the study evaluates complex multicomponent programs, protein-rich diets, and branched-chain amino acid supplementation. By addressing the nexus of nutrition and cognitive health, this review contributes to understanding viable interventions for promoting cognitive well-being in aging populations.

## 1 Introduction

As the World Health Organization reports, the pace of population aging is accelerating. In 2022, there were 771 million people aged 65+ years globally, accounting for almost 10% of the world’s population (1). This segment has been growing at an increasing rate, and it is expected to be about a quarter of the global population by 2,100.

Several factors have contributed to this trend. Life expectancy has meaningfully increased in the Western world due to healthcare, nutrition, and technology advances. However, the increasingly aging population has posed substantial challenges to healthcare and social service systems and will impede future developments. Evidently, aging seniors require more healthcare attention and long-term care, straining upon resources and public finances (2). Therefore, the primary social interest is in taking active steps to maintain mental and physical health in the population and to preclude or delay the accompanying dementia dependence.

In recent years, several comprehensive reviews on the topic of dementia prevention have been published. A systematic review by Williams et al. (3) concluded that no sufficient evidence exists to recommend interventions preventing cognitive decline and dementia. However, since then, understanding of the pathological processes that culminate in clinical dementia has meaningfully advanced. Several clinical trials of potential preventive interventions have been completed and eventually published, with more underway and being planned.

In addition, terminology and classification related to cognitive deficiency have changed in recent years. Dementia itself is referred to now as “major neurocognitive disorder” in accordance with the fifth edition of the Diagnostic and Statistical Manual of Mental Disorders 2013 update (4). Of note, dementia is an umbrella term that describes a broad group of symptoms associated with cognitive impairment interfering with basic daily functioning (5).

Several medical conditions lead to major neurocognitive disorder; however, Alzheimer’s disease (AD) is the most common cause, accounting for about 70% of cases (6). AD is a progressive neurological disorder marked by cognitive decline, memory loss, and behavioral changes (7). Although the precise cause remains unclear, it is thought to result from a complex interplay of genetic, environmental, and lifestyle factors (8). Besides the distinct hallmarks, the disease exhibits systemic abnormalities and metabolic brain aberrations (9).

Despite over a century of multinational efforts, no effective cure for AD has been found. Current treatment mainly involves medications that manage symptoms and slow down dementia progression to some extent, but only in specific cases (10). Numerous drugs were tested, but none prevent dementia. The FDA-approved monoclonal antibodies targeting aggregated forms of amyloid-β, Aducanumab and Lecanemab by Biogen, remain controversial (11). They cause brain swelling and bleeding (12), cerebral edema, microhemorrhages, and other adverse events (13). Accordingly, the concept of using monoclonal antibodies recognizing protofibrils to treat AD is still questionable.

As a consequence of repetitive failure to find efficient treatment strategies, some alternative approaches have been proposed, including non-pharmacological interventions such as cognitive stimulation (14), regular exercise (15), and a healthy lifestyle (16), which were proven to support overall brain health.

The main goal of our report is to inform the public and scientific society about novel approaches to maintaining brain health with aging via appropriate diets. This review examines the current evidence on efficient dietary interventions reliably preventing aging-associated cognitive decline and dementia. In addition, we intend to inform future efforts to develop new strategies and suggest novel approaches for improving the quality of life of the elderly population. While the current evidence has not yet matured enough to support a serious public health campaign focused upon the widespread adoption of such interventions, our work suggests at least a novel topic for extensive public and scientific discussion of those interventions.

## 2 Alzheimer’s disease as a complex metabolic disorder

AD is an intricate illness, comprising interconnected pathological events like inflammation, neurovascular issues, bioenergetics, and systemic processes. Beyond the classic hallmarks, it exhibits systemic and brain-specific metabolic abnormalities detectable at molecular and biochemical levels.

Mounting evidence supports the view of AD as a disorder characterized by seriously affected peripheral organs and blood in a similar magnitude as the brain (17). Of note, the features and scope of AD-associated metabolic abnormalities resemble the pathology observed in obese and diabetic patients (18, 19). It is well-established that diabetes causes severe oxidative stress, brain insulin resistance, and, eventually, cognitive impairment (20). Some groups have even suggested that AD is a sub-type of diabetes—type 3 diabetes (21, 22). Even though this term is not officially accepted and is not recognized by National Health Organizations, it reflects a considerable overlap between AD and diabetes mellitus at molecular and biochemical levels. Moreover, elderly diabetic patients develop wide-ranging abnormalities that resemble classic AD pathology (23).

There are indications that metabolic failure is an early and fundamental event in AD pathogenesis (24). Dementia-associated metabolic deficits include glycolysis dysfunction, glucose metabolism dysregulation, and pentose phosphate pathway impairment. Accordingly, several interventions normalizing metabolic aberrations have been suggested to prevent dementia development (25, 26).

Of note, the brain is an extremely metabolically active organ. It consumes about 20% of body oxygen while at rest, but it represents just 2% of the body’s mass (27). On the other hand, it is susceptible to oxidative imbalances (28). Therefore, minor metabolic deviations in the brain lead to severe oxidative stress and neuronal malfunction. Accordingly, some groups have hypothesized that increased levels of diet antioxidants may help prevent dementia development. Recent work by May Beydoun et al. (29) analyzed blood samples from more than 7,000 people between the ages of 45 and 90. The acquired data were linked to existing databases that tracked participants over 16 years to establish an association between incidents of all-cause dementia and serum levels of antioxidant vitamins and carotenoids, which were found inversely correlated.

Several clinical trials evaluated the therapeutic potential for antioxidants in AD; however, with no conclusive results (30). Nevertheless, there are indications that a combination of antioxidants and nutrient-rich diets may tackle AD pathogenesis (31).

In addition to metabolic aberrations associated with systemic pathology, brain hypoperfusion due to atherosclerosis, endothelial dysfunction, infections, brain injury, autoimmune disorders, and other diseases can lead to oxidative stress in the CNS (32). Of importance, these factors are preventable or at least efficiently treatable in the Western world.

Recently collected evidence on the incidence and prevalence of dementia indicates a trend of decline in high-income countries that successfully manage cardiovascular diseases and diabetes mellitus (33), which offers hope that public health interventions may be effective in preventing cognitive decline and dementia as well.

## 3 Is it possible to prevent dementia via lifestyle change?

According to the dominant theory, AD-associated changes in the CNS occur many years prior to the first symptoms of the disease appear (34). Consequently, there is a possible therapeutic window to preclude or, at least, delay debilitating memory loss and other dementia symptoms.

On the other hand, advanced stages of major neurocognitive disorder are not curable. Severe brain atrophy accelerates over the course of the disease as a subject progresses from cognitively normal to demented (35). Thus, there is no treatment to substitute the lost brain matter and recuperate its normal function with skills and memories acquired during a lifetime and vanished at once but forever; and the only option to tackle the disease is to prevent its development and preserve mental health and sanity.

Several dietary approaches have been studied for their potential impact on AD. While none can guarantee prevention or cure, specific diets have shown promise in supporting brain health and potentially reducing the risk of cognitive decline. Intermittent fasting has been gaining attention for its potential neuroprotective effects in the context of AD (36). Animal studies showed that this type of fasting may have a positive impact on neurodegenerative processes (37). It was associated with improvements in synaptic adaptations leading to amelioration of cognitive deficits (38). These findings indicated that intermittent fasting may play a potential role in preventing and reducing the symptoms of AD.

Of note, laboratory mice are typically fed a nutritionally balanced diet that does not resemble the typical diet of people in Western cultures (39). Hence, to investigate the effects of a diet on age-related changes in the brain, some researchers have developed a diet for mice that imitates the diets commonly consumed in Western societies (40). This diet was shown to lead to astrogliosis and microglia activation in the wild-type mice and cause an even more severe phenotype in AD model mice, where glial response was associated with increased plaque burden in the hippocampus (40, 41). Thus, animal studies point to the significant effect of diets on brain function and possible prevention of age-related dementia and AD.

The notion of diet’s impact on dementia prevention is not new, with various studies highlighting the influence of nutrition on aging brain function. Current clinical research explores non-traditional interventions to delay disease progression. Given the disease’s complexity, it’s believed that a combination of drugs and individualized treatment strategies, considering factors like background and clinical status, offers the most promising results (42). Still, the problem of the multiagent intervention efficacy evaluation is the paramount obstacle.

Randomized controlled trials (RCTs), considered the gold standard in evidence generation (43), offer the strongest evidence, albeit demanding substantial resources and time. RCTs work well for testing single-agent or single-intervention approaches. However, addressing the complex physiological processes of cognitive decline necessitates multi-layered methods that are challenging to assess via standard RCTs.

In this context, the Cognitive Impairment and Disability (FINGER) trial is the first RCT showing that it is possible to prevent cognitive decline using a multidomain lifestyle intervention among at-risk elderly patients (44). This trial underscores the importance of addressing multiple risk factors as a robust strategy to safeguard the brain, enhance overall health, reduce the risk of other chronic illnesses, and improve functioning.

FINGER, an ongoing RCT in Finland, runs until 2030 and is coordinated by the Finnish Institute of Health and Welfare in collaboration with the Karolinska Institute (Sweden). It assesses the impact of a multidomain intervention in delaying cognitive impairment and disability in the elderly. Over a thousand participants aged 60–77 were randomized into intervention or control groups. The intervention covered nutritional guidance, physical exercise, cognitive training, social activities, and managing vascular and metabolic risk factors. The control group received regular health advice. Dietary guidance adhered to Finland’s national recommendations, emphasizing fish, fruits, vegetables, and whole grains, aligning with the “healthy Nordic diet” for brain health. Interestingly, this diet bears similarities to the Mediterranean diet, which is also associated with reduced dementia risk (45).

Briefly, the FINGER intervention included consumption of fruits and vegetables above 400 g/d, whole-grain cereal products, low-fat milk and meat, sucrose intake of less than 50 g/day, vegetable margarine instead of butter, and fish consumption of at least two portions per week. Weight correction was personalized based on individual factors, and all participants, including the control group, were advised to take at least 10 μg/day of supplemental vitamin D. Notably, adherence to this diet predicted improved global cognition and enhanced executive function.

In this trial, the exercise intervention encompassed strength training alongside aerobic and balance exercises with progressively increasing intensity. Cognitive training included group meetings and computer-based individual sessions, targeting domains prone to age-related decline: episodic memory, working memory, processing speed, and executive function. Management of metabolic and vascular risk factors followed national evidence-based guidelines and involved meetings with a study nurse and physician for measurements, counseling, and lifestyle management.

After 2 years of complex intervention, cognition improved by approximately 25% in the experimental group compared to the control group. The performances improved in all cognitive sub-domains irrespective of sociodemographic, socioeconomic factors, or other baseline characteristics. Moreover, APOE-ε4 carriers also had clear cognitive benefits from the intervention (44). The researchers conclude that dietary changes initiated during the intervention are related to changes in executive function in 2 years. However, a long-term diet appears to be more influential for global cognition.

An extended follow-up study is currently in progress to assess the long-term effects of the intervention. The Finnish Institute for Health and Welfare also initiated a project to implement the study findings into everyday practice. This project includes identifying at-risk individuals and guiding them toward adopting a healthy aging lifestyle.

In our view, efficiently delaying age-related cognitive decline necessitates maintaining a healthy lifestyle during midlife, prior to the onset of impairment symptoms. It is also important to recognize that for elderly individuals with memory deficiencies and other signs of aging-related changes, altering established unhealthy behaviors is more challenging than for healthy adults. The abovementioned epidemiological study by Oliver M. Shannon et al. (45) on Mediterranean diet adherence association with dementia risk supports our view. The study analyzed incidents of all-cause dementia cases in 60,298 participants from the UK Biobank, followed for more than 9 years. The authors demonstrate that higher adherence to a Mediterranean diet is strongly associated with lower dementia risk, independent of genetic risk. Despite the non-interventional nature of the study, its results further underline the importance of diet in dementia prevention.

In fact, an RCT was performed in Spain to examine whether the Mediterranean diet influences cognitive function (46). More than 400 cognitively healthy volunteers (mean age 66.9 years), at high cardiovascular risk were enrolled into the nutrition intervention trial from October 2003 through December 2009. In the analysis of composite cognitive change, individuals in the intervention groups showed significantly better cognitive outcomes than those in the control group, which points to dementia preventive features of the Mediterranean diet in the older population. Of note, the Mediterranean diet is rich in fruits, vegetables, whole grains, fish, and healthy fats. It has been extensively studied for its potential anti-inflammatory and antioxidant properties and is associated with various health benefits, including cardiovascular health, possibly due to its anti-inflammatory and antioxidant properties (47, 48).

## 4 Promising dietary supplements to enhance cognition

Following the lack of effective treatment in recent years, several herbal remedies, dietary supplements, and “medical foods” have been actively marketed and promoted as memory enhancers and possible dementia treatments. Some even claim they can delay or prevent AD and other dementias. Despite mounting evidence suggesting that a healthy diet may reduce dementia risk, no single ingredient, food, vitamin, or supplement has been proven to prevent or cure AD.

Of importance, in the US, “medical food” can legally refer only to food that treats a condition with a distinctive nutritional requirement (49). However, major neurocognitive disorder does not have such a requirement. Accordingly, no food can be marketed or labeled as medicinal for dementia.

We have mentioned above indications that antioxidant-rich food may help protect the brain from free radicals. Commonly consumed colorful fruits and vegetables, such as berries, leafy greens, broccoli, tomatoes, etc., are rich in various polyphenols and other efficient antioxidants (50). In the developed world, colorful fruits and vegetables are available all year round. However, despite significant improvements in the quality and variety of food, it became common to consume poly-vitamins and other supplements on a regular basis. Some specialists mention dramatically changing sociodemographic conditions, rising away-from-home food expenditures, and significantly deteriorating food preparation skills in modern Western society as the main reasons to substitute vitamins (51). Consequently, worldwide use of nutritional supplements has increased dramatically over the recent decades (52). However, some nutrition studies suggest that the intake of vitamin supplements may be needless and even harmful (53–55). Thus, this subject remains controversial.

In this context, several epidemiologic studies investigated whether B-vitamin status is associated with cognitive decline and cognitive function generally. Some established an association between B-vitamin status indicators and AD or other dementias. Circulating levels of vitamins inversely correlate with the risk of cognitive impairment (56, 57). Consequently, some groups proposed to monitor serum B12 and folate concentration in the elderly as a preventive AD procedure (58).

Several RCTs have evaluated the hypothesis that B-vitamin supplementation prevents cognitive decline. One of the first double-blind, controlled vs. placebo studies by Fioravanti et al. (59) assessed the efficacy of folic acid supplement in aged patients with abnormal cognitive decline. Notably, patients treated with folic acid for 60 days significantly improved memory and attention efficiency compared to a placebo group. A more recent large-scale RCT with a 2-year-long intervention established that daily supplementation of folic acid and vitamin B12 promotes improvement of cognitive functioning in the elderly, particularly in immediate and delayed memory performance (60). Of note, reliable sources of B-vitamins include leafy greens, cereals, legumes, dairy products, eggs, poultry, and fish. Some of these products are main components of the Mediterranean diet.

Omega-3 fatty acids from plant and fish sources are considered a capable non-medical alternative to improve brain functions and halt dementia progression. This assumption is predicated upon results from several epidemiological studies and preclinical pieces of research. A recent large-scale epidemiological study established that within older adults, regular fish oil supplementation is strongly associated with a lower risk of incident all-cause dementia (61). These findings suggest that habitual use of fish oils may be beneficial for preventing dementia in clinical practice.

Freund-Levi et al. (62) performed a randomized double-blind trial to show that administration of omega-3 fatty acid in patients with mild to moderate AD does not delay the rate of cognitive decline. However, positive effects were observed in a group of patients with very mild AD, which points to a possible therapeutic window to halt the AD development. Even though another randomized placebo-controlled trial provided no convincing evidence for the efficacy of omega-3 supplements in the treatment of AD (63), a meaningful interest in testing omega-3 fatty acids formulations to treat dementia drives more complex large-scale investigations. Of importance, the effect of omega-3 fatty acid supplementation appears to be influenced by baseline homocysteine, suggesting that adequate B vitamin status is required to obtain the beneficial effects of omega-3 on cognition (64).

In this context, it is noteworthy to mention the recent findings related to a patented combination of nutrients, including uridine monophosphate, choline, omega-3 fatty acids, phospholipids, vitamin C, vitamin E, selenium, vitamin B6, vitamin B12, and folic acid marketed as Souvenaid. The results of a three-year-long, randomized, double-blind, placebo-controlled trial in 311 people with prodromal AD have been published recently. Souvenaid, taken as a 125-ml breakfast drink, significantly slowed the decline of clinical and other measures related to cognition, function, brain atrophy, and disease progression (65). Recently completed post-hoc analysis proved a statistically significant effect of the treatment upon the rate of cognitive decline (66) and indicated positive effects of long-term multinutrient intervention in prodromal AD.

Notably, fatty fish such as salmon, mackerel, and sardines; walnuts, flaxseeds, and chia seeds are reliable sources of omega-3 fatty acids. These foods are the main components of healthy Nordic and Mediterranean diets.

## 5 A high-protein diet may protect the aging brain

Accruing evidence from metabolomics studies indicates significant deficits in the levels of some amino acids in the risk groups for dementia development. Subsequently, some authors suggested that a diet high in protein may confer some protection against AD.

Of importance, in the healthy organism, the amino acid composition of physiological fluids is in a dynamic equilibrium state, and any long-term imbalance may lead to or reflect a disease. The plasma free amino acid (PFAA) profile is known to reflect nutritional status (67) and may have the potential to predict changes in cognitive function. A recent report by Takeshi Ikeuchi et al. (68) presents the results of a two-year interim analysis of a three-year longitudinal study following patients with mild cognitive impairment (MCI). Fasting plasma samples were collected from participants. Two years after blood collection, they were divided into two groups: those who remained with MCI and those who converted to AD, based on standard clinical assessment of cognitive function. The baseline PFAA profile was compared between the two groups, and additional analysis based on APOE-ε4 allele possession was conducted. Remarkably, plasma concentrations of all nine essential amino acids were lower in the AD-convert group. However, three branched-chain amino acids (BCAAs): valine, leucine, and isoleucine, showed the most significant differences. Of note, the logistic regression model adjusted for confounding factors such as gender, body mass index, and APOE-ε4 allele possession also achieved statistical significance. Though, in the stratified analysis, differences in plasma concentrations of BCAAs and histidine were more pronounced in the APOE-ε4-negative group. The authors conclude that the PFAA profile, and mainly decreases in the levels of BCAAs and histidine, is associated with AD development. They further suggest that the PFAA profile is an independent AD development risk indicator.

It is worth noting that PFAA profiles have been associated with the risks of developing diabetes and cancer (69). One of the causes for the amino acid profile alterations may be the metabolic shift resulting from insulin resistance. However, in older adults, other factors may significantly influence the amino acid plasma composition. The nutritional status and personal dietary habits affect the amino acid profile and emerge as essential components of chronic degenerative diseases, including AD (70).

The aging organism’s digestion, absorptive capacity, and assimilation of various nutrients gradually decline (71). Reduced absorption of key nutrients, including proteins, is also frequent due to atrophic gastritis, which is very common in the elderly (72). Likewise, metabolic rate tends to decline with age, and so does general physical activity. In addition, numerous comorbidities requiring pharmaceutical interventions may further compromise the dysbalanced nutrient status (73).

Population-based studies indicate that the risk of dementia is significantly reduced among individuals adhering to a diet with a high percentage of caloric intake from protein sources. In one study, the researchers investigated the associations between the percentage of daily energy derived from carbohydrates, fat, and protein and the risk of MCI. In this case, more than 1,000 elderly participants (70–89 years old) were followed over a median of 3.7 years. The results clearly point to a diet with a relatively high caloric intake from carbohydrates and a low intake from fat and proteins as increasing the risk of dementia (74).

Kaplan et al. (75) demonstrated that 774 kJ of pure whey protein intake enhanced memory performance in healthy elderly. Of importance, in contrast with glucose and fat, protein was the only macronutrient to influence the rate of forgetting on the paragraph recall test at 15 min.

Rokicki et al. (76) applied functional magnetic resonance imaging and a battery of neuropsychological tests to assess the effect of carnosine/anserine cocktail consumption on episodic memory and resting state network connectivity in healthy participants (aged 40–78). After 3 months of treatment, the carnosine/anserine group demonstrated significantly better verbal episodic memory and decreased connectivity in the default mode network compared to the control group.

An objective cross-sectional study was performed to determine the relationship between dietary protein and fiber intake, and plasma and brain Aβ levels in a cohort of cognitively normal aged adults (77). In this study, the authors utilized advanced positron emission brain tomography and focused just on cognitively normal individuals to avoid potential differential misclassification bias in dietary intake due to progressive cognitive impairment. The results suggest a negative correlation between protein intake and brain Aβ burden, highlighting the protective impact of increased dietary protein consumption. The authors speculate that high-protein diets may be beneficial for maintaining brain health.

One recent longitudinal Harvard study by Tian-Shin Yeh et al. (78) evaluated the dietary habits and health conditions of more than 77,000 men and women who were followed for over 20 years. The extended follow-up period is a major strength of this study that allowed the critical windows of exposure to be captured and minimized the reverse causation impacts. The results indicate that in comparison with carbohydrates, consumption of protein is associated with lower chances of developing cognitive decline later in life. The authors found a strong correlation between protein intake and dementia risk. They demonstrate that every 5% of calories coming from animal protein instead of carbohydrates corresponds to a reduction in the dementia risk by 11%. Furthermore, for every 5% of calories from plant protein instead of carbohydrates, there is a 26% reduction in the risk of dementia developing later in life.

A longitudinal study by Gao et al. examined the association of protein intake from different sources with cognitive decline (79). The authors analyzed the dietary habits of 3,083 participants aged 55–93 years. Their cognition was assessed in 1997, 2000, 2004, 2006, and 2015. Diet intake was evaluated using weighing methods in combination with 24-h dietary recalls for three consecutive days at each survey. The results indicate that increasing animal protein consumption in a population with plant-dominant diets prevents cognitive decline.

Of importance, while these representative observational studies do not establish a causal link between increased protein consumption and brain protection, they do suggest that proteins play a crucial role in maintaining cognitive function.

There were attempts to decipher the biological mechanisms responsible for the effects of amino acid supplementation on neurocognitive function. Hideaki Sato et al. (80) used a rodent model (55–63-week-old mice) fed a low-protein diet for 2 months to demonstrate a decline in memory and an increase in agitation levels. The observed behavioral abnormalities correlated with the levels of neurotransmitters, such as γ-aminobutyric acid, glutamate, glycine, dopamine, norepinephrine, serotonin, and aspartate. Notably, supplement of essential amino acids reversed cognitive impairments. The authors conclude that low-protein intake causes low blood amino acid levels and results in neurotransmitters’ deficiency.

Furthermore, research conducted in mice showed that exposure of aged animals to young blood reverses age-related impairments in hippocampal-dependent learning and memory, suggesting that factors present in young blood, such as proteins, may have rejuvenating effects on the aging brain (81). A more recent study by Zhao et al. (82) in AD mice evidenced young plasma treatment-related improvement in memory, and reduction of tau, Aβ pathologies, and neuroinflammation.

## 6 A putative role of branched-chain amino acids in dementia development

Animal and plant foods containing all nine essential amino acids in adequate proportions, such as meat, eggs, quinoa, and soy, are considered complete proteins (83). Essential amino acids are crucial for the proper functioning and growth of the human body and must be obtained through diet. BCAAs are amino acids possessing an aliphatic side chain with a branch. Three proteinogenic BCAAs, valine, leucine, and isoleucine, are all essential amino acids.

BCAAs are abundant in mammals and comprise about one-third of all amino acids present in humans (84). Skeletal muscles particularly contain a substantial amount of BCAAs that serve as central structural components and nitrogen accumulators (85). In addition to their structural role, BCAAs represent key signal molecules regulating pancreatic insulin production and protein synthesis by controlling the translation initiation phase in the skeletal muscles and liver.

Pioneering experiments in rats with radiolabeled amino acids demonstrated that BCAAs readily cross the blood–brain barrier (86). Furthermore, BCAAs uptake by the brain surpasses all other amino acids (87). In the mammalian brain, BCAAs fulfill several critical functions. They are involved in the protein synthesis, energy production, and metabolism of key neurotransmitters (88).

Several complex studies attempted to correlate the brain, cerebrospinal fluid (CSF), and plasma amino acid composition with strengths of memory acquisition and recall, and other cognitive functions. Basun et al. (89) collected CSF and plasma from subjects with dementia of Alzheimer’s type and healthy volunteers with no clinical or family history of dementia. Notably, individuals with dementia demonstrated a substantial reduction in the CSF concentration of valine compared to healthy controls.

A study by González-Domínguez et al. (90) utilized advanced gas chromatography coupled with mass spectrometry to prove a significant reduction in the levels of valine in the serum of AD patients compared to healthy controls. Consequent studies in murine AD models involving deep profiling of the brain and the plasma metabolome have also found significantly disturbed BCAAs metabolism (91–93).

Advanced metabolomics methods are widely used to detect peripheral metabolic changes in the aging population and AD patients. Several groups tried to correlate plasma metabolic changes with CSF pathology markers, imaging features, and cognitive performance. Toledo et al. (94) performed an extensive cross-sectional study with 730 participants. The authors analyzed fasting serum samples to identify the key AD-associated metabolic and disease-progression-related changes. Of importance, plasma valine levels were shown to negatively correlate with the rate of cognitive decline. Moreover, the coefficient for valine negatively correlated with objective ventricular volume changes in these patients. On the other hand, an increase in valine concentration was associated with a significantly decreased risk of AD.

Another extensive study with 22,623 participants by Tynkkynen et al. (95) utilized nuclear magnetic resonance and mass spectrometry to profile blood metabolites. Ten metabolites related to a high incidence of dementia were identified. Notably, lower levels of all three BCAAs were strongly associated with an increased risk of dementia and AD.

Considering the existing epidemiological and metabolomics data, a double-blind, randomized, placebo-controlled trial was conducted by Sizuki et al. (96). The researchers examined the effects of seven selected essential amino acids, including all three BCAAs (used in the ratios associated with the brain influx rate), on cognitive and psychosocial functions in healthy adults aged 55 years or older, who are the target group for dementia prevention. Notably, ingesting the amino acid cocktail for 12 weeks significantly improved attention, cognitive flexibility, and psychosocial functioning. Accordingly, the authors conclude that amino acid supplements may prevent cognitive decline in old age.

In this context, an intriguing question is emerging. Do the short-term improvements in cognitive performance observed in the groups with amino acid supplementation translate into delay or slowing of cognitive decline and reduced risk of dementia? In our view, future research on amino acids supplementation or just protein-rich diets may be of the most value if it can clarify whether the effects upon memory function and other cognitive performances are limited to the group of patients at risk. Another emerging question concerns the diagnostic and prognosis value of plasma amino acids profiling. We believe a higher-risk population may be selected based on lower measured BCAAs levels.

## 7 Conclusion

In the context of a rapidly aging global population, preserving cognitive function and preventing age-related cognitive decline have emerged as crucial public health imperatives. Notably, despite sustained international endeavors, the persistent lack of a disease-modifying drug for AD presents substantial medical and social challenges. While there has been commendable progress in unraveling the fundamental pathophysiology of cognitive decline and senile dementia, the absence of efficient medical and social solutions remains a pressing issue.

The evolving landscape of research and healthcare is responding to this challenge by putting forth new approaches and strategies aimed at preventing or arresting the development and manifestation of these cognitive disorders. The urgency of addressing this gap in medical intervention has spurred innovative thinking and collaborative efforts, encompassing a spectrum of disciplines, from neuroscience to public health. As we navigate this complex terrain, the quest for effective solutions continues, driven by the collective commitment to enhance the quality of life for an aging global population facing the specter of cognitive decline.

The landscape of dementia-related nutritional research is notably promising, marked by a plethora of interventional studies, both completed and ongoing, that portend the imminent arrival of novel and crucial insights. While preclinical studies and clinical trials may have left some aspects inconclusive, the overall trajectory is optimistic, with an abundance of encouraging results that underscore the efficacy of potential interventions. Recent strategies proposed for dementia prevention exhibit a nuanced and inclusive approach, signaling a departure from traditional perspectives and addressing the pathology beyond its conventional hallmarks. Emerging interventions and evolving strategies offer hope for effective solutions to dementia challenges. As scientific knowledge and innovative strategies advance, the field is positioned for transformative breakthroughs to mitigate these debilitating conditions.

Of note, several alternative diet approaches have been proposed and tested preclinically and clinically (97). One such approach is the ketogenic diet, which has been studied for its potential benefits in various health conditions, including AD and other neurodegenerative diseases (98). Ketone bodies were found to reduce neuroinflammation, improve synaptic maintenance, and reduce brain β-amyloid deposition (99). While limited human studies have shown the potential benefits of a ketogenic diet in AD, more extensive and rigorous clinical investigations are needed to establish its efficacy and safety. Of importance, the ketogenic diet, with or without fasting, can lead to short-term weight loss, but it may have harmful side effects like ketoacidosis. People, particularly those with type II diabetes or other serious health conditions, are advised to consult their physicians before starting this diet (100). Moreover, the long-term effects of a ketogenic diet on overall health should also be considered.

In summary, the prevention of aging-related cognitive impairment demands innovative strategies that specifically address prominent risk factors prevalent in older age, many of which are shared with other chronic disorders. Recognizing the interrelated nature of these risk factors provides a foundation for developing holistic approaches targeting the complex challenges associated with cognitive decline in elderly. By employing tactics considering the overlapping influences of various risk factors, we can advance a more comprehensive and nuanced approach to promoting cognitive well-being in older individuals.

As a compelling clinical application, we propose that the cost-effective monitoring of plasma levels of BCAA represents a potent strategy for screening substantial segments of the aging population, facilitating the early identification of individuals susceptible to age-related health challenges. By leveraging advances in analytical techniques, such as mass spectrometry, to precisely quantify BCAA concentrations in plasma, this approach offers a streamlined and accessible means of large-scale screening. The incorporation of BCAA monitoring into routine health assessments holds the potential to proactively pinpoint individuals at risk, enabling timely intervention and personalized strategies to promote healthy aging. This innovative method aligns with the current trend in precision medicine and preventive healthcare, underscoring its viability as an efficient and powerful tool in geriatric health management.

Our belief in the significant influence of diet upon dementia management aligns with the growing recognition that while it may not reverse the condition, adopting a healthy diet, particularly in midlife, can play a pivotal role in supporting brain health and overall well-being. This perspective underscores the proactive nature of interventions, emphasizing the potential impact of lifestyle choices on cognitive outcomes.

In this context, the multifaceted FINGER intervention, characterized by its intricate integration of nutritional guidance, targeted physical exercise regimens, cognitive training protocols, engaging social activities, and comprehensive management of vascular and metabolic risk factors, emerges as a highly promising and contemporary approach. We propose the imperative acceptance and global implementation of a parallel initiative, encompassing diverse social and age demographics. This initiative should extend beyond merely addressing identified at-risk individuals, aiming to guide individuals across various societal strata in adopting a health-promoting lifestyle conducive to successful aging. The overarching objective is to afford every individual the opportunity to proactively steer toward a life replete with vitality and cognitive engagement until the point of physiological decline. Such a global initiative aligns with the paradigm shift toward preventive healthcare strategies and holds the potential to significantly enhance public health on a worldwide scale.

In the developed world, an ongoing public health campaign specifically targeting the protection of aging brains and the preservation of mental health is a positive development. This concerted effort reflects a broader understanding that extends beyond pharmacological solutions, recognizing the importance of behavioral and gastronomic protocols. The prospect of preventing cognitive decline and senile dementia without solely relying on drugs provides a hopeful trajectory for the future. In the coming years, the convergence of research, public awareness, and lifestyle interventions may pave the way for effective strategies that empower individuals to take charge of their cognitive health and contribute to a collective effort in averting the impending calamity of dementia.

## Author contributions

BP: Conceptualization, Methodology, Writing – original draft, Writing – review & editing. AS: Conceptualization, Funding acquisition, Methodology, Project administration, Supervision, Writing – original draft, Writing – review & editing.
